# Supplementary material for: Anti-CD49d Ab treatment ameliorates age-associated inflammatory response and mitigates CD8+ T-cell cytotoxicity after traumatic brain injury
Source: J Neuroinflammation. 2024 Oct 19;21:267. doi: 10.1186/s12974-024-03257-7 (PMC11491007; doi:10.1186/s12974-024-03257-7)
Supplement: Supplementary file 1 — Additional file 1. Figure S1. aCD49d Ab treatment reduced CD8+ T cells in the aged brains. A. i-ii. Representative aCD49d Ab lymphocyte depletion in the aged brains is shown. iii-iv. aCD49d Ab treatment specifically reduced CD8+ T cells in the aged brains but not CD4+ T cells. B. i. Representative flow plots showing the gating for infiltrating and proliferating CD45+CD11b- and T cells within the brains. C. i-ii. Representative EdU+ proliferating cells in the brain are shown. D. Quantifications of i. infiltrating, ii. proliferating, iii. total T cells within aged brains 3 days post TBI. All data are shown as the mean ± SEM, 2-way ANOVA with Tukey’s multiple comparisons test for A. Student’s t-test for D. n = 5-9/group for A, *p < 0.05, **p < 0.01, ***p < 0.001. n = 3–4/group for D. Figure 2. No effect of aCD49d Ab was seen in the behavioral performances of young mice post TBI. Results of A. rotarod indicated by time spent on accelerating wheels (s) and B. Y maze indicated by %alteration score. Data are from two independent experiments. All data are shown as the mean ± SEM, 2-way ANOVA with Tukey’s multiple comparisons test. n = 8-10/group for A and B. Figure 3. A. Representative flow plots showing the gating for the measurement of different immune cells in the blood. B. gating for CD49d expression measurement. C. Correlation of CD49d expression in different immune cells in the blood with age. All data are shown as the mean ± SEM, Pearson’s correlation analysis, n=15. *p < 0.05, **p < 0.01. Figure 4. Multiplex cytokine analysis in aged and young mice at 7 days post injury. Levels of plasma cytokines including A. IL12p70 and B. IL1β. n =3-4/group, *p < 0.05. All data are from one independent experiment. Data are shown as the mean ± SEM, 2-way ANOVA with Tukey’s multiple comparisons test. Figure 5. Representative flow plots showing the gating for A. CD4+ T cells in the brains and their expression of transcription factors for Th1 (T-bet), Th2 (GATA3), and Th17 (RORgt) re [file 12974_2024_3257_MOESM1_ESM.docx]

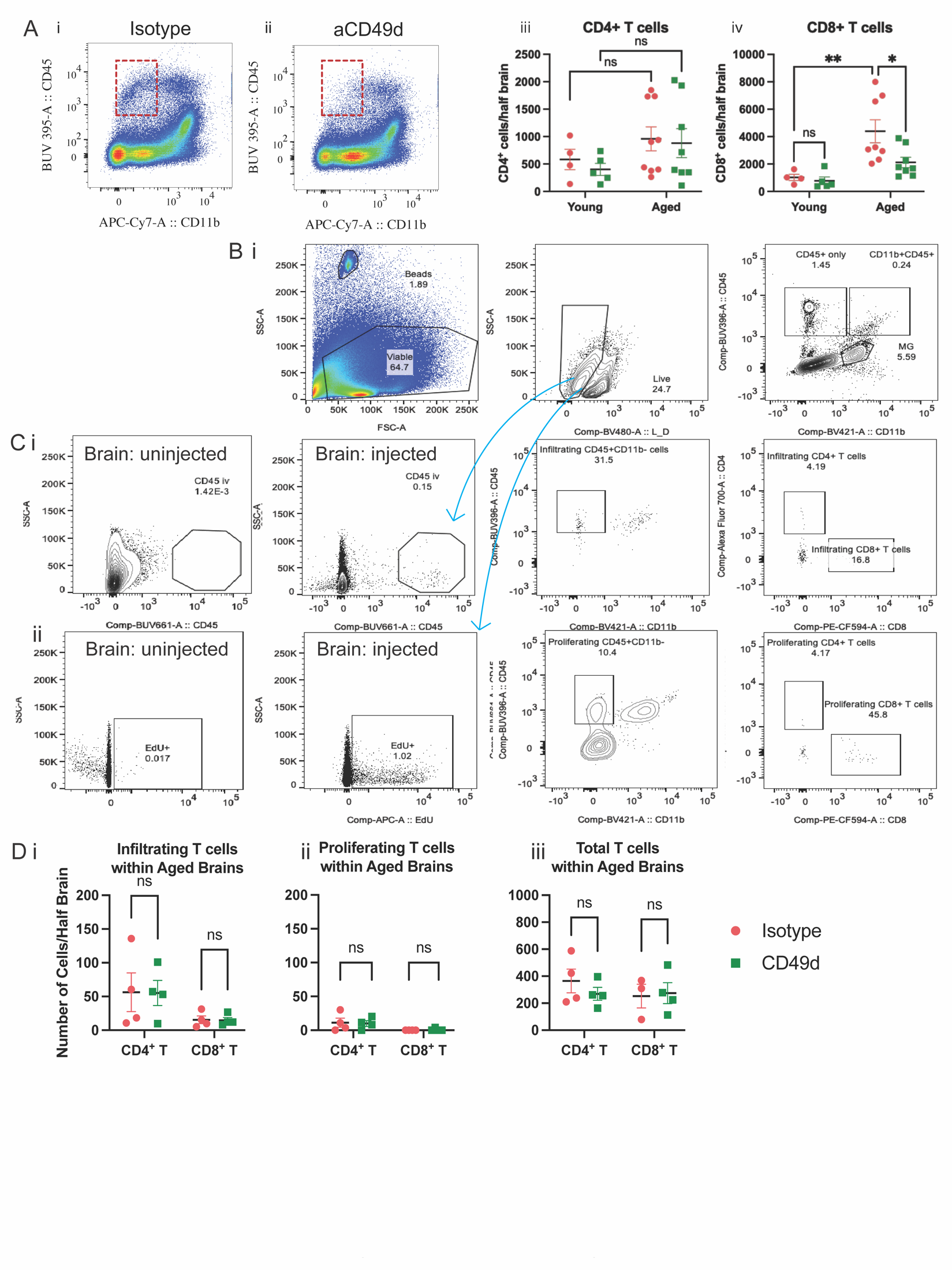


Supplement Figure 1. **aCD49d Ab treatment reduced CD8+ T cells in the aged brains.** A. i-ii. Representative aCD49d Ab lymphocyte depletion in the aged brains is shown. iii-iv. aCD49d Ab treatment specifically reduced CD8+ T cells in the aged brains but not CD4+ T cells. B. i. Representative flow plots showing the gating for infiltrating and proliferating CD45^+^CD11b^-^ and T cells within the brains. C. i-ii. Representative EdU^+^ proliferating cells in the brain are shown. D. Quantifications of i. infiltrating, ii. proliferating, iii. total T cells within aged brains 3 days post TBI. All data are shown as the mean ± SEM, 2-way ANOVA with Tukey’s multiple comparisons test for A. Student’s t-test for D. n = 5-9/group for A, *p < 0.05, **p < 0.01, ***p < 0.001. n = 3-4/group for D.


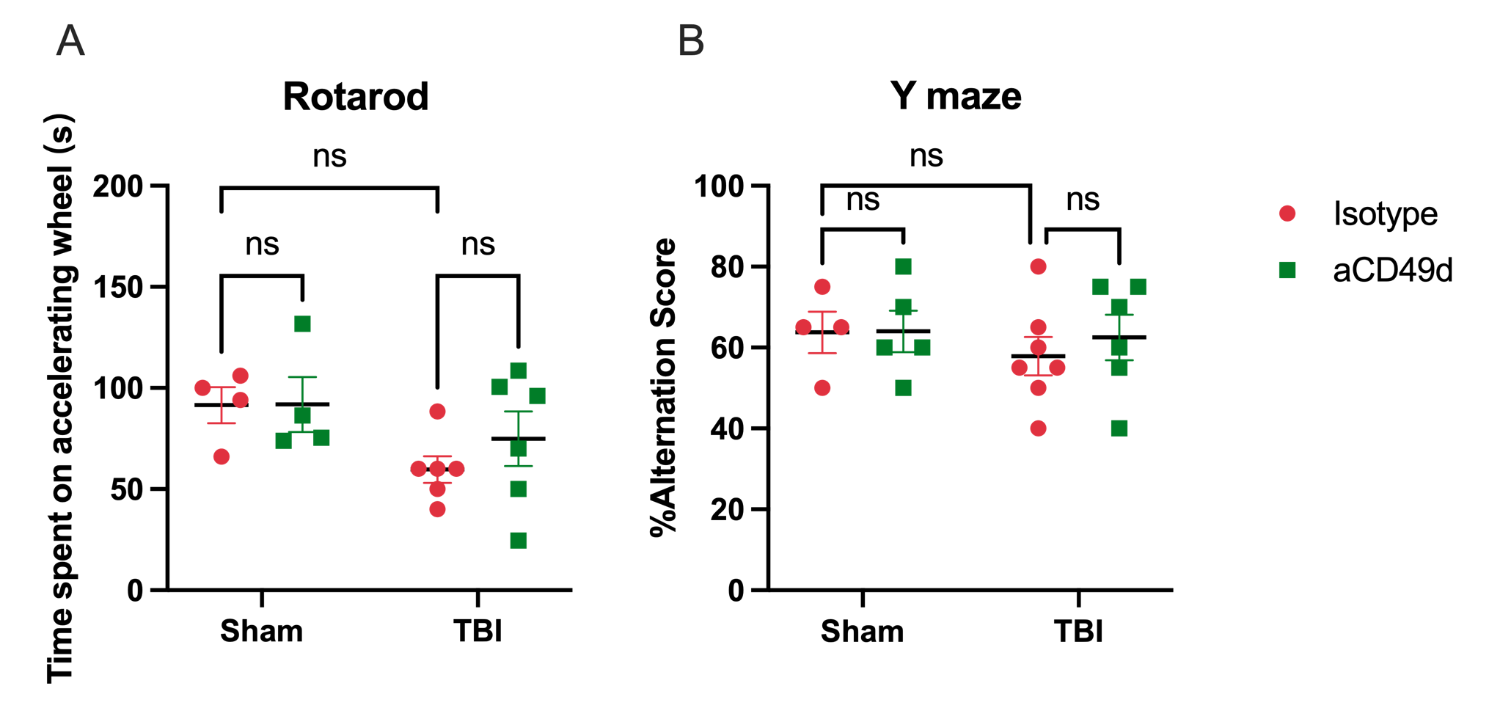


Supplement Figure 2. **No effect of aCD49d Ab was seen in the behavioral performances of young mice post TBI**. Results of A. rotarod indicated by time spent on accelerating wheels (s) and B. Y maze indicated by %alteration score. Data are from two independent experiments. All data are shown as the mean ± SEM, 2-way ANOVA with Tukey’s multiple comparisons test. n = 8-10/group for A and B.


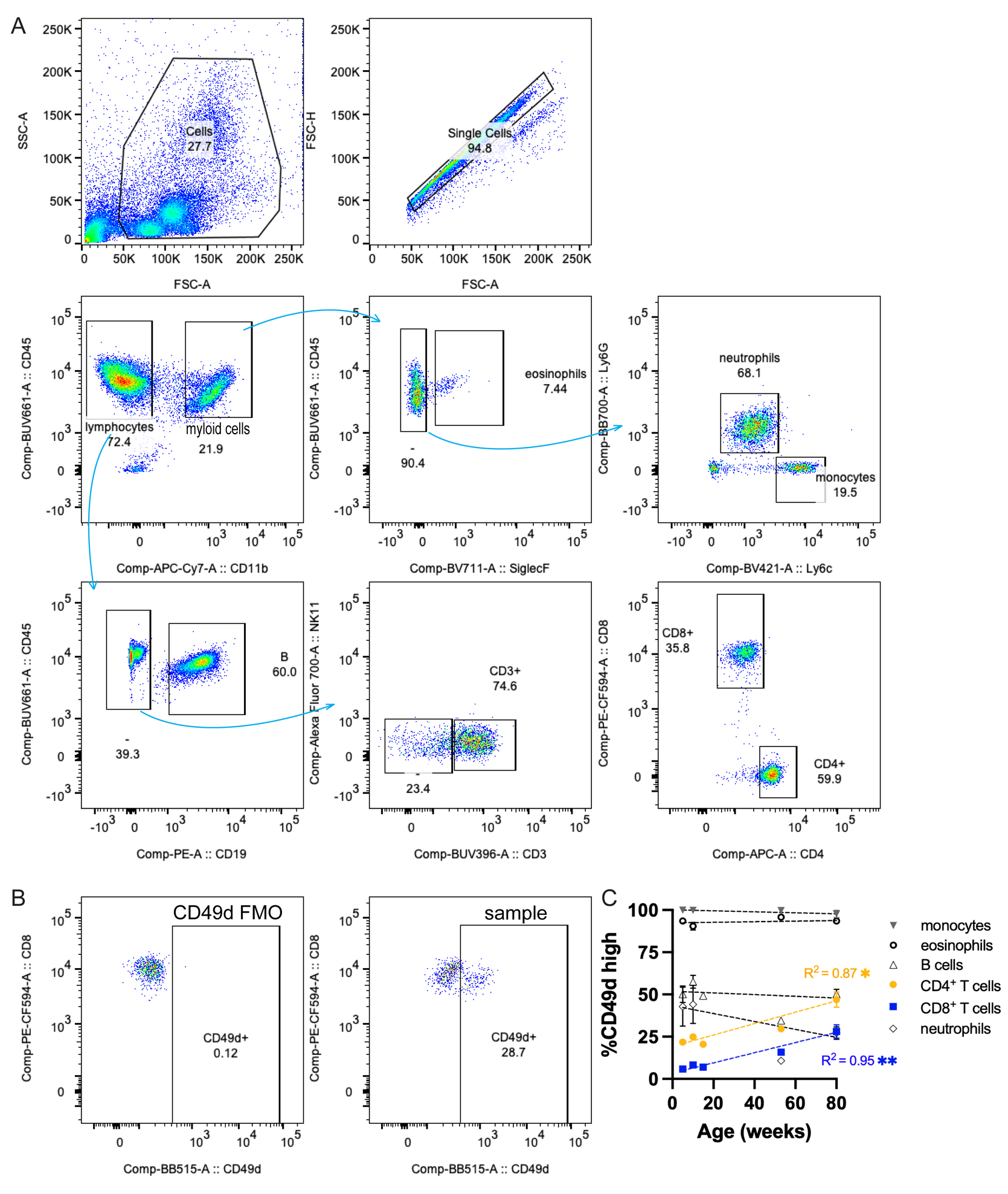


Supplement Figure 3. A. Representative flow plots showing the gating for the measurement of different immune cells in the blood. B. gating for CD49d expression measurement. C. Correlation of CD49d expression in different immune cells in the blood with age. All data are shown as the mean ± SEM, Pearson’s correlation analysis, n=15. *p < 0.05, **p < 0.01.


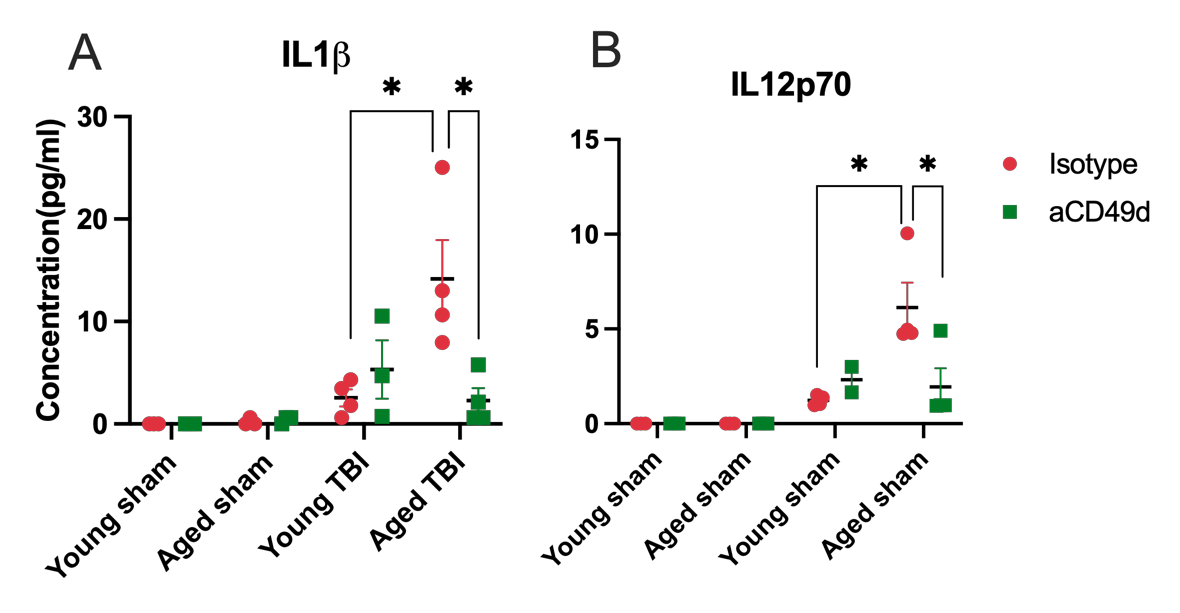


Supplement Figure 4. **Multiplex cytokine analysis in aged and young mice at 7 days post injury**. Levels of plasma cytokines including A. IL12p70 and B. IL1β. n =3-4/group, *p < 0.05. All data are from one independent experiment. Data are shown as the mean ± SEM, 2-way ANOVA with Tukey’s multiple comparisons test.


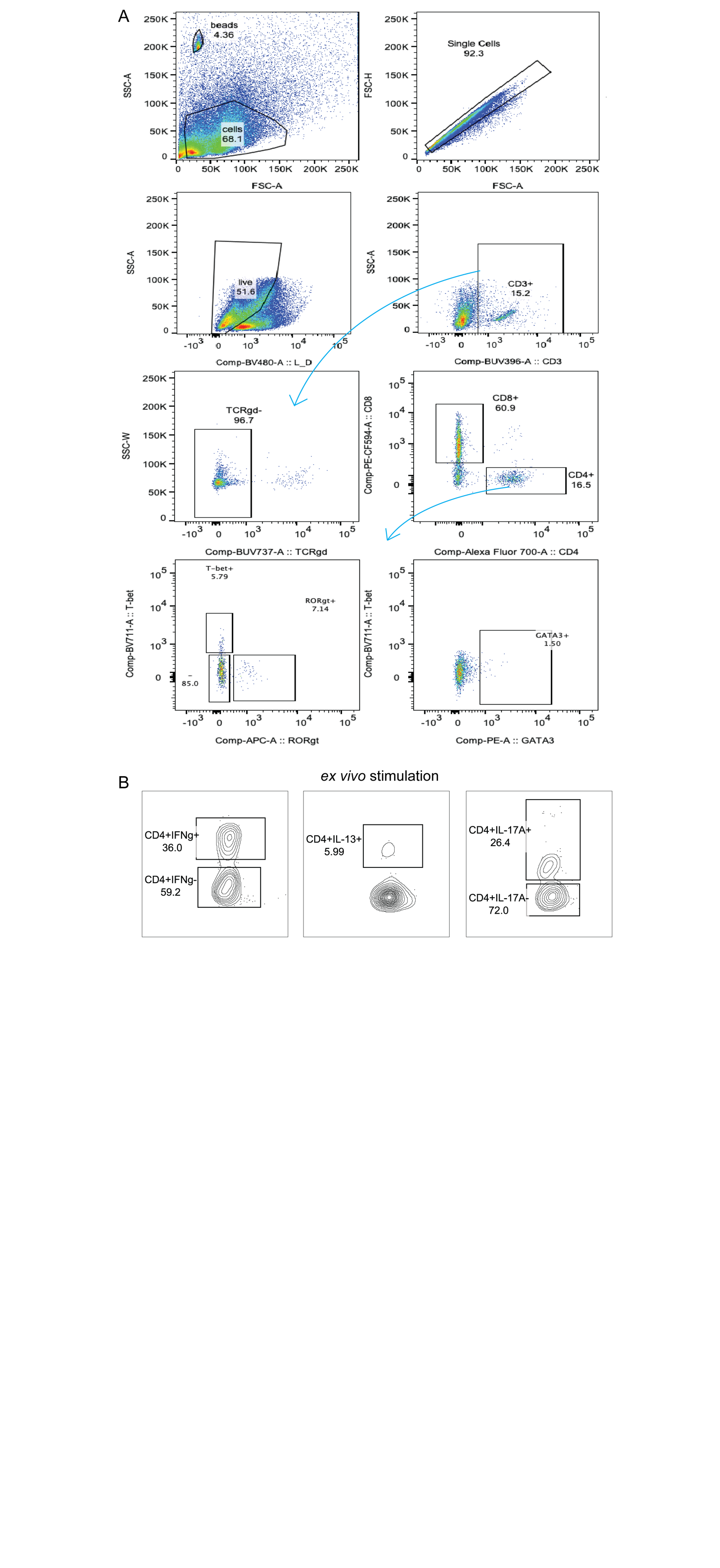


Supplement Figure 5. Representative flow plots showing the gating for A. CD4+ T cells in the brains and their expression of transcription factors for Th1 (T-bet), Th2 (GATA3), and Th17 (RORgt) response. B. in a separate experiment, brain cells were *ex vivo* stimulated with PMA/ionomycin and incubated with golgi-plug containing brefeldin A for four hours followed by measurement of IFNg, IL-13, and IL-17A expression in CD4+ and CD8+ T cells.


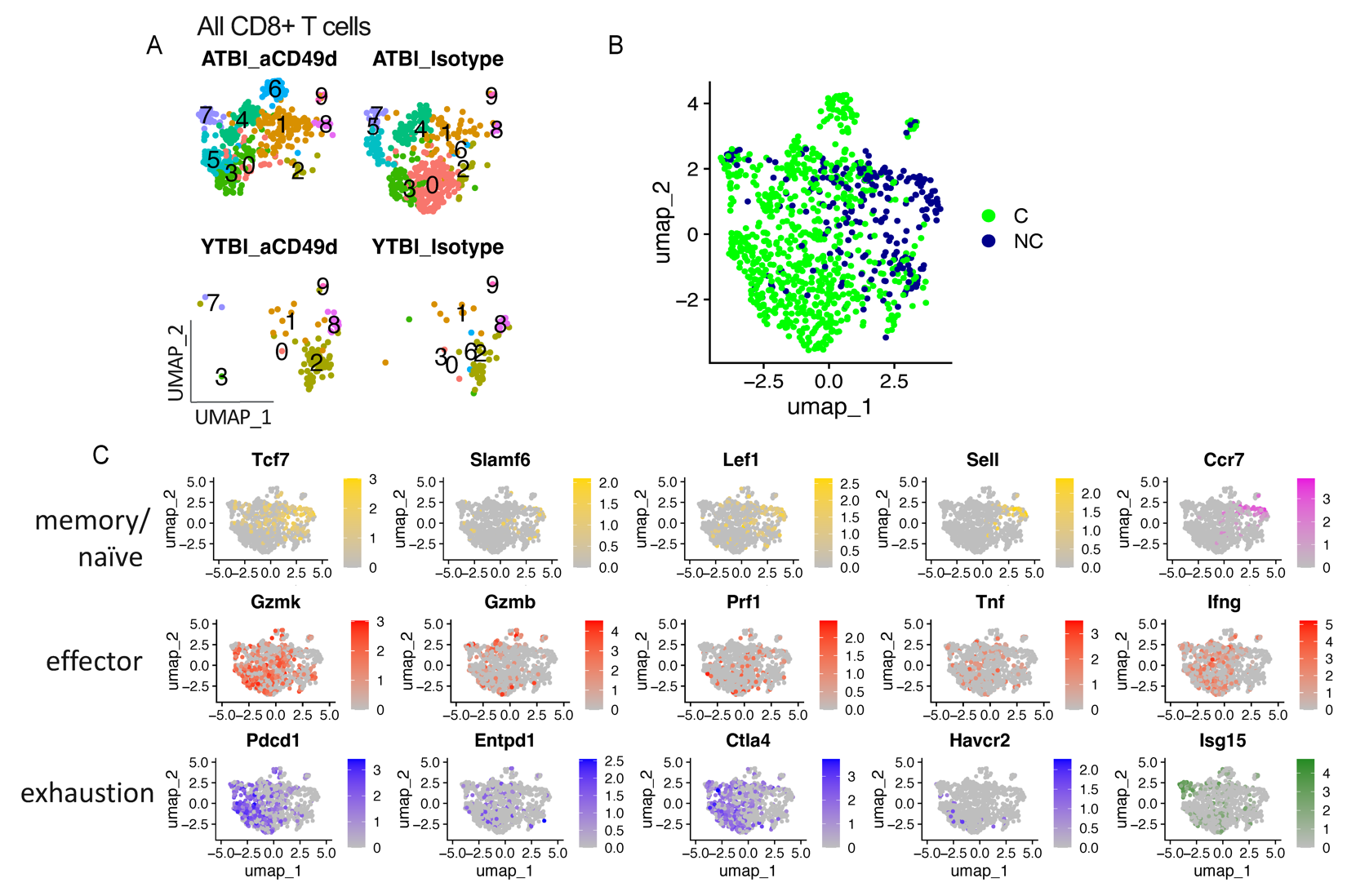


Supplement Figure 6. A. UMAP showing unsupervised clustering across samples. B. single-cell TCR analysis overlaid on UMAP projections showing distribution of CD8+ T cell clonality. C. Feature plots depicting various T cell differentiations states reveals that brain T cells from aged TBI mice had two pools of activated CD8+ T cells: dysfunctionally and functionally activated.


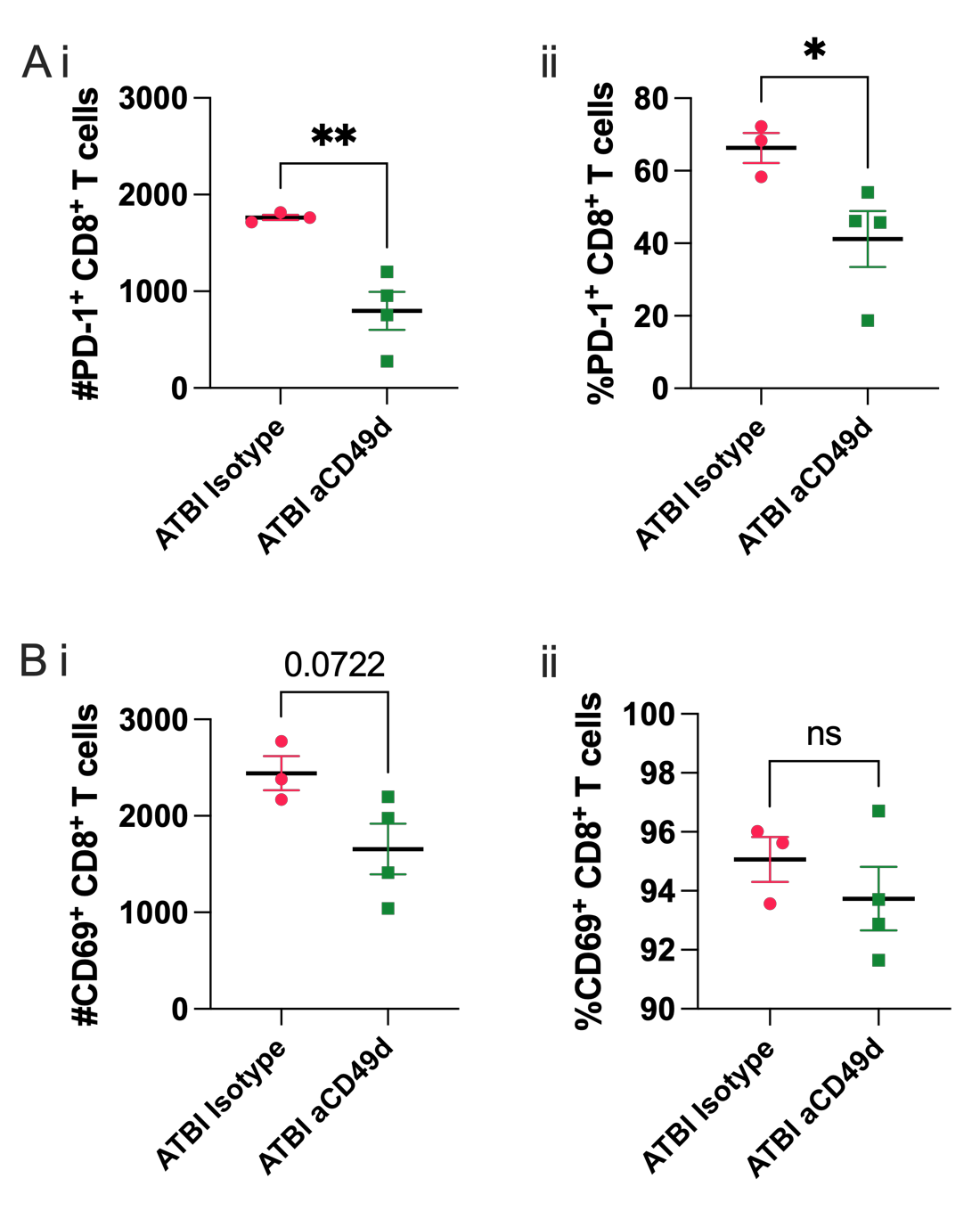


Supplement Figure 7. A. i Quantification of PD-1+ and ii. % PD-1+ CD8+T cells in all CD8+ T cells in aged mouse brains with isotype vs aCD49d Ab treatment. B. i Quantification of CD69+ and ii. CD69+ CD8 T+ cells in all CD8+ T cells in aged mouse brains with isotype vs aCD49d Ab treatment. n =3-4/group, *p < 0.05, **p<0.01. All data are from one independent experiment. Data are shown as the mean ± SEM, 2-way ANOVA with Tukey’s multiple comparisons test.


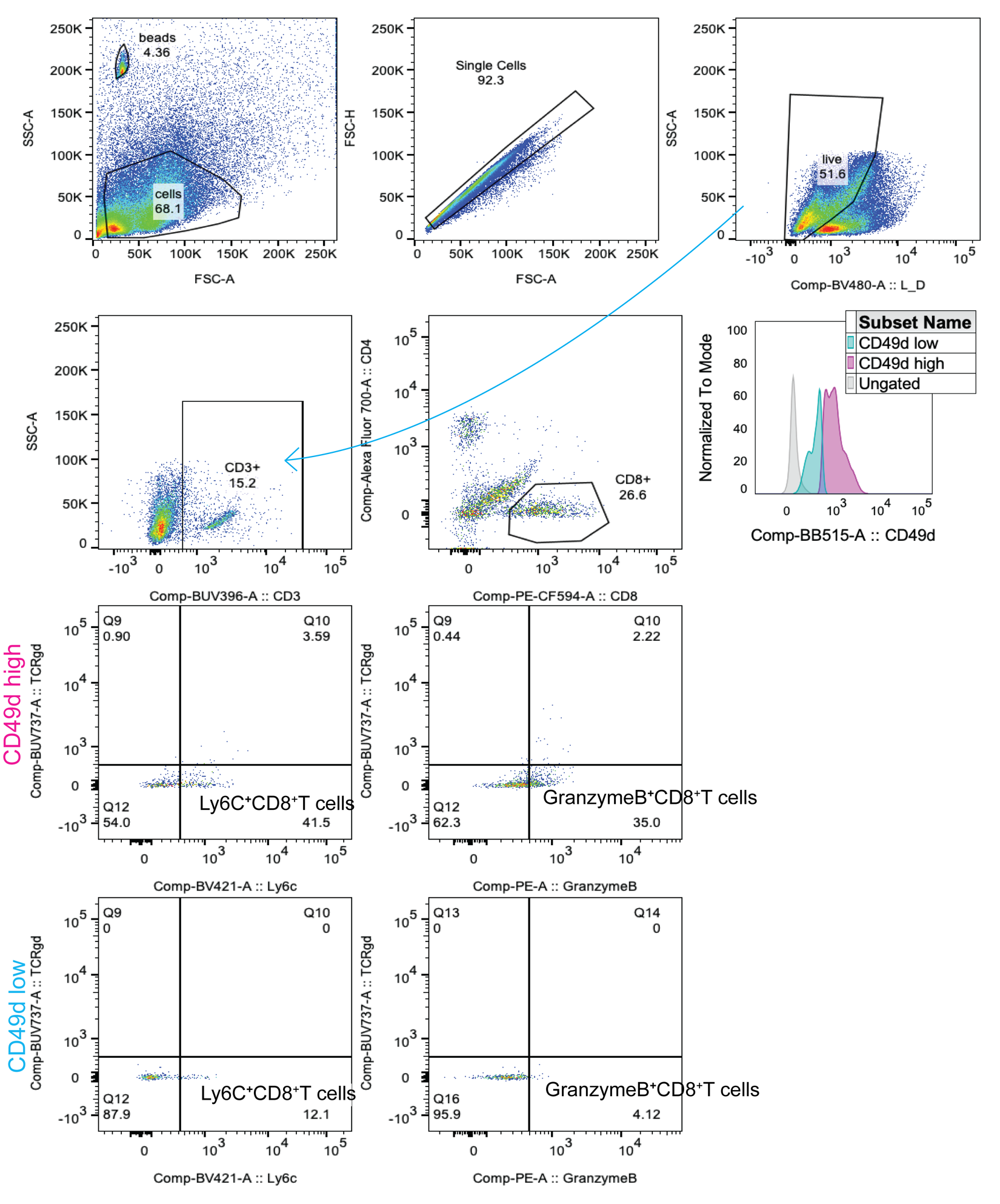


Supplement Figure 8. Representative flow plots showing the gating for Ly6C+ and Granzyme B+ CD49d high vs CD49d low CD8+ T cells within the brains.

Supplement Figure 9. Quantifications of A. CD64+ macrophages, B. Ly6c+ monocytes and C. CD45^dim^CD11b+ in both young and aged mouse brains 2 months post TBI. Data are from two independent experiments and shown as the mean ± SEM, 2-way ANOVA with Tukey’s multiple comparisons test. n=2-6/group, *p < 0.05, **p < 0.01.


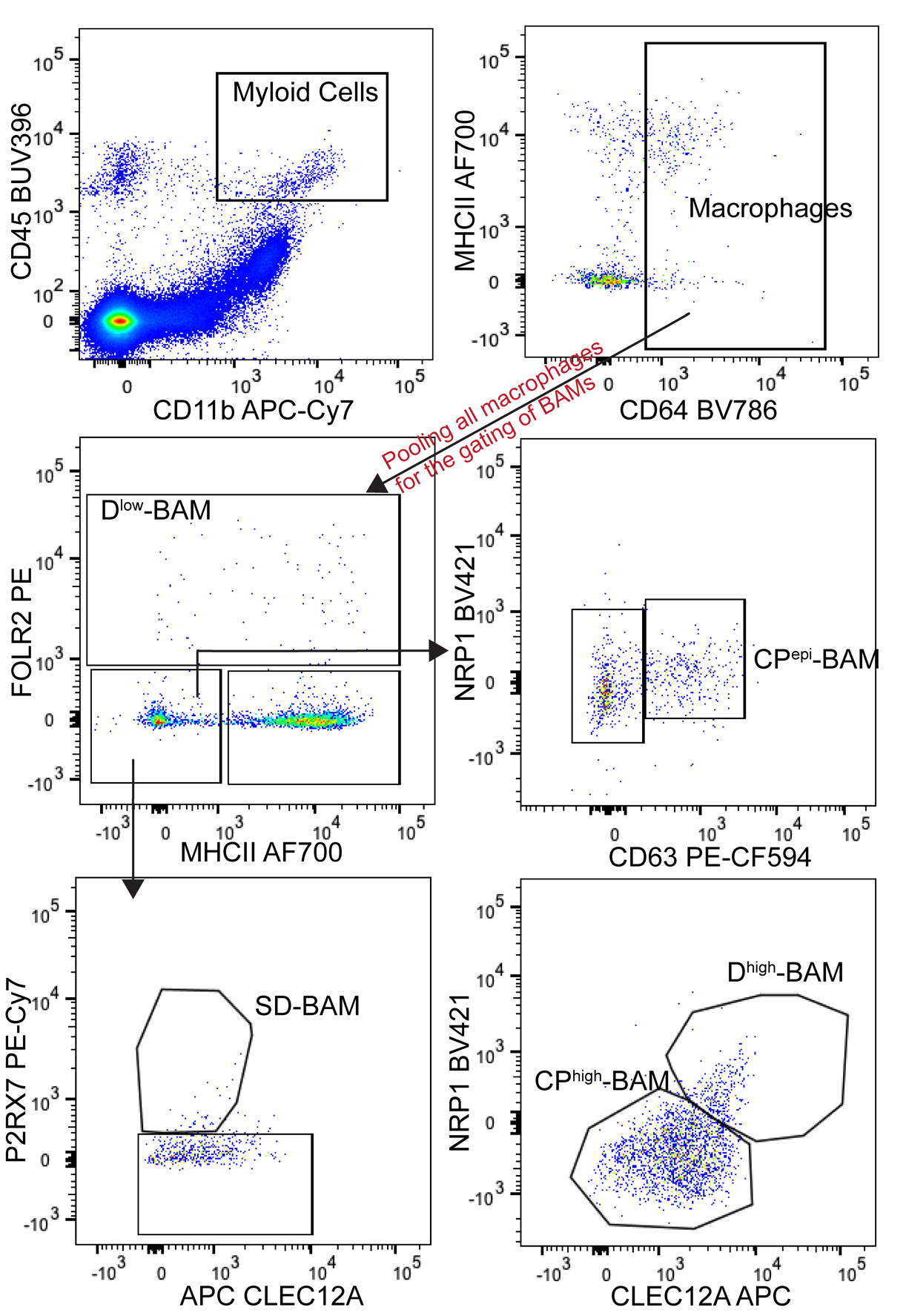


Supplement Figure 10. Gating strategy for tissue-specific brain resident macrophages or BAM. CD45+CD64+ macrophages from all samples were pooled (concatenated using FlowJo) first followed by being further gated on FOLR2 and NRP1 for dural BAM (D-BAM), MMR and P2RX7 for subdural BAM (SD-BAM), and CD63 for choroid plexus BAM (CP-BAM).

**
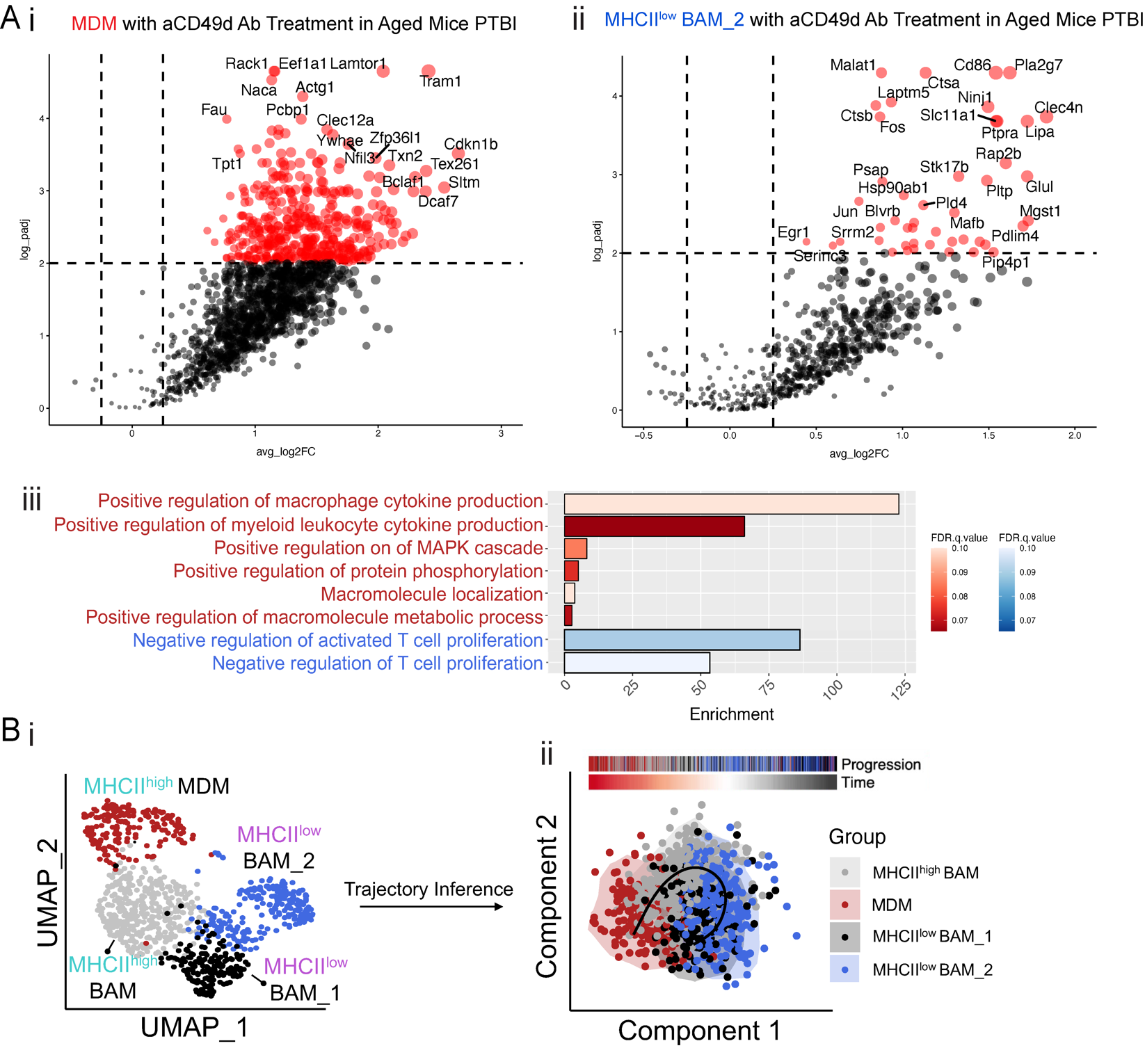
**

Supplement Figure 11. Volcano plots displaying genes that are DE (adjusted P<0.01, log2(FC)>0.25) between i. MDMs with aCD49d Ab versus isotype treatment and ii. BAMs with aCD49d Ab versus isotype treatment in aged mice two months post TBI. No downregulated DEs are found in both groups.
